# Divergent Electrophysiologic Effects of Sacubitril in Digitalis- and Pinacidil-Related Shortened Repolarization: Experimental Evidence for Harmful Effects of Digitalis Glycosides

**DOI:** 10.3390/pharmaceutics17030338

**Published:** 2025-03-06

**Authors:** Christian Ellermann, Carlo Mengel, Julian Wolfes, Felix K. Wegner, Benjamin Rath, Florian Reinke, Lars Eckardt, Gerrit Frommeyer

**Affiliations:** Department of Cardiology II (Electrophysiology), University Hospital Münster, Albert-Schweitzer-Campus 1, 48149 Münster, Germany

**Keywords:** sacubitril, arrhythmia, pinacidil, ouabain, digitalis, short QT

## Abstract

**Background**: Recent studies reported an abbreviation of cardiac repolarization induced by sacubitril. Thus, the purpose of this study was to evaluate the electrophysiologic effects of sacubitril in the presence of drugs that shorten the QT interval. **Methods and Results**: 25 rabbit hearts were retrogradely perfused. After generating baseline data, hearts were allocated to two groups. In the first group (*n* = 12), the I_K,ATP_ opener pinacidil (1 µM) significantly reduced action potential duration at 90% of repolarization (APD_90_), QT intervals and effective refractory periods (ERP). Additional administration of sacubitril (5 µM) slightly reduced APD_90_. The digitalis glycoside ouabain (0.2 µM) significantly shortened repolarization duration and refractory periods. Additional infusion of sacubitril abbreviated repolarization duration and ERP. Ventricular vulnerability was assessed by delivering premature extra stimuli and burst stimulation. Significantly more ventricular arrhythmias occurred with pinacidil (26 episodes vs. 5 episodes under baseline conditions, *p* < 0.05). Additional sacubitril treatment had no significant proarrhythmic effect (24 episodes). Ouabain alone did not provoke ventricular arrhythmias (6 episodes vs. 3 under baseline conditions, *p* = ns) whereas additional sacubitril treatment significantly increased the occurrence of VT episodes (29 episodes, *p* < 0.01). **Conclusions**: Sacubitril abbreviates cardiac repolarization in ouabain-pretreated hearts. While sacubitril had no proarrhythmic effect in the presence of pinacidil, the combination of sacubitril and ouabain amplified the arrhythmic risk. The underlying mechanism is a further abbreviation of refractory periods and cardiac repolarization that facilitate ventricular arrhythmias. These findings add further evidence to the proarrhythmic capacity of digitalis glycosides in the presence of other drugs that influence cardiac repolarization.

## 1. Introduction

Sacubitril in combination with valsartan is established for pharmacological therapy of heart failure with reduced ejection fraction [1]. Previous studies [2] demonstrated an abbreviation of cardiac repolarization induced by sacubitril which might, along with other drugs that influence cardiac electrophysiology, facilitate arrhythmias [3,4]. In a previous study [2] using a Langendorff-perfused rabbit heart model, sacubitril shortened cardiac repolarization duration in the presence of a stable spatial dispersion of repolarization. In the context of simulated long QT syndrome type 2, sacubitril exhibited antiarrhythmic properties by reducing spatial heterogeneity, thereby suppressing torsades de pointes tachycardia. Notably, sacubitril also decreased the inducibility of atrial fibrillation in this model [2]. The underlying molecular mechanisms of its repolarization-abbreviating effect have not been fully elucidated, but are likely related to complex interactions with cardiac ion channels (specifically I_Ks_, I_Kr_, I_K,ATP_, I_Na,L_, or I_Ca_) [2]. The risk for drug-induced arrhythmias is particularly pronounced in patients with a reduced repolarization reserve, such as those with heart failure. Not only since the first description of the short QT syndrome [5], an abbreviation of the QT interval has been regarded as a potential arrhythmic indicator [6]. In the general population, a corrected QT interval ≤ 300 ms is associated with a 2.6-fold increased risk of death [7]. Drug-induced QT interval shortening could be a potential harbinger of proarrhythmia [8] and might be underreported as the first manifestation of a short QT interval can be ventricular fibrillation, which often leads to sudden cardiac death. In contrast, drug-induced QT prolongation typically results in torsade de pointes tachycardia that are often transient and not necessarily fatal, making it more widely recognized [8]. Though not widely acknowledged, it is possible that the combination of drugs that abbreviate repolarization increases the likelihood of ventricular arrhythmias potentially leading to syncope and sudden cardiac death.

In short QT syndrome, the abbreviation of cardiac repolarization, shortening of refractory periods and amplification of spatial dispersion of repolarization facilitate the occurrence of ventricular arrhythmias [9]. In experimental models, these effects can be mimicked by pinacidil, an I_K,ATP_-opener, which causes a substantial abbreviation of action potential duration, amplification of spatial dispersion of repolarization, and thereby increases ventricular vulnerability [10]. A possible clinical cause of acquired short QT syndrome is the treatment with digitalis glycosides [11,12]. Digitalis glycoside intoxication can trigger delayed afterdepolarizations and thus induce polymorphic ventricular tachycardias [13,14,15]. In several randomized trials, treatment with digoxin was associated with an increased risk of sudden death [16,17]. Still, digitalis is recommended for rate control of atrial fibrillation in heart failure patients [1].

Thus, the aim of this study was to assess the electrophysiologic effects of sacubitril in the presence of two agents that abbreviate cardiac repolarization, pinacidil and the digitalis glycoside ouabain.

## 2. Materials and Methods

All experimental protocols were approved by the local animal care committee (Landesamt für Natur, Umwelt und Verbraucherschutz Nordrhein-Westfalen, Germany; file number: 81-02.05.50.21.004) and were carried out in accordance with the ARRIVE guidelines and the Guide for the Care and Use of Laboratory Animals published by the US National Institutes of Health (NIH Publication No. 852-3, revised 1996). Hearts were not randomized since they served as their own control. The sample size calculation was based on previous studies from our group with similar assumed effect sizes. No animals were excluded from this study.

The technique of the Langendorff perfusion has been reported previously [2]. In brief, 25 hearts of female New Zealand white rabbits were retrogradely perfused. The AV node was ablated by compressing the interatrial septum with surgical tweezers to enable programmed stimulation. Hearts were perfused at a constant pressure (90 mmHg) and flow (52 mL/h) using a warmed and oxygenated (95% O_2_, 5% CO_2_) modified Krebs–Henseleit Buffer (NaCl 118 mM, NaHCO_3_ 24.88 mM, D-glucose 5.55 mM, KCl 4.70 mM, Na-pyruvate 2 mM, CaCl_2_ 1.80 mM, KH_2_PO_4_ 1.18 mM, MgSO_4_ 0.83 mM). To obtain monophasic action potentials, eight specifically designed catheters were positioned, seven epicardially around the heart and one endocardially in the left ventricle. A 12-lead ECG was recorded.

**Figure 1:**
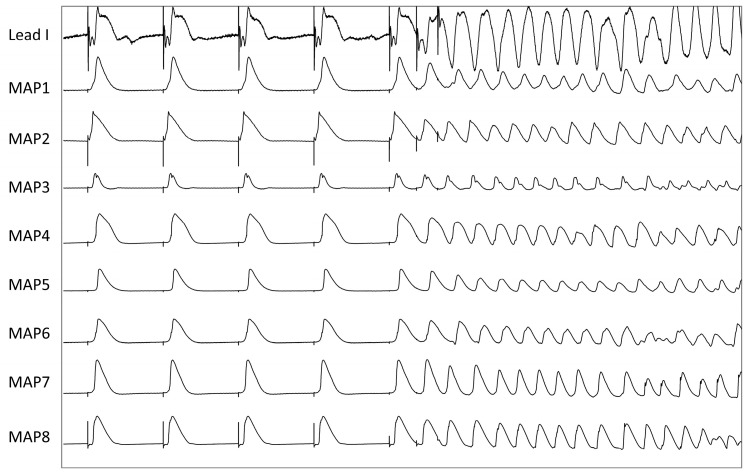
Induction of a polymorphic ventricular tachycardia after delivering two short-coupled extrastimuli (S3) under the influence of pinacidil (MAP = monophasic action potential).

**Figure 2:**
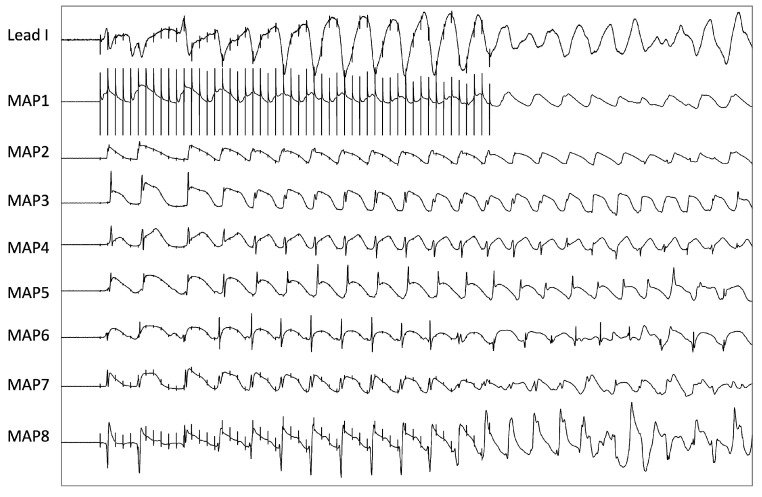
Example of a polymorphic ventricular tachycardia induced by burst pacing under combined infusion of ouabain and sacubitril (MAP = monophasic action potential).

Having completed the experimental setup, a pacing protocol was performed to obtain cycle length-dependent (900–300 ms) QT intervals and action potential durations that were measured between the fastest upstroke of the phase 0 of the action potential and 90% of repolarization (APD_90_). Premature extrastimuli (S_2_ and S_3_) were delivered after a train of seven stimuli at a basic cycle length of 900–300 ms. Thereby, effective refractory periods (ERP) were determined and the inducibility of ventricular arrhythmias was assessed (Figure 1). Additionally, burst pacings were utilized to provoke ventricular arrhythmias (Figure 2). The spatial dispersion of repolarization was determined by the difference between the longest and shortest APD_90_. Post-repolarization refractoriness (PRR) was defined as the difference between ERP and APD_90_.

After generating baseline data, 25 hearts were divided up into two groups. The first group (*n* = 12) was treated with the I_K,ATP_ opener pinacidil (1 µM). In the second group, the digitalis glycoside ouabain was administered (*n* = 13, 0.2 µM). Having completed the experimental protocol with the aforementioned drugs, both groups were additionally perfused with sacubtril (5 µM) and the protocol was repeated again.

### 2.1. Drugs

Pinacidil, a drug previously used to treat arterial hypertension [18], is now primarily used in experimental settings. Therefore, the pinacidil concentration employed in this study is mainly derived from previous experimental studies. Specifically, our group has utilized pinacidil in several prior studies, yielding robust and reproducible electrophysiological parameters consistent with those seen in short QT syndrome (e.g., [19]).

Ouabain, a digitalis derivative no longer approved for clinical use in the US, is particularly suited for studying acute effects due to its intravenous formulation and rapid onset of action. It is now mainly used in experimental research. The ouabain concentration used in this study (0.2 µM) is derived from previous experiments that provided reproducible results [15,20,21].

In clinical practice, the median maximum plasma concentration of sacubitril ranges from approximately 5.1 µM at a dose of 200 mg sacubitril/valsartan twice daily to around 2.3 µM at a dose of 100 mg twice daily [22]. For this study, we used a concentration of 5 µM, which corresponds to the plasma level observed with a 200 mg dose of sacubitril/valsartan twice daily, considering poor metabolizers [22]. Of note, this concentration had the most pronounced abbreviating effect on cardiac repolarization in a previous study [2]. Importantly, no significant proarrhythmic effects were observed at this concentration.

### 2.2. Statistics

Action potentials and electrograms were recorded on a multi-channel recorder and digitalized at a rate of 1 kHz with a 12-bit resolution. Variables are displayed as mean ± standard deviation. Statistical analyses were conducted employing SPSS Statistics for Windows (version 24.0). Drug effects on APD_90_, QT interval, spatial dispersion of repolarization and effective refractory periods were analyzed using Wilcoxon signed rank test. *p* values < 0.05 were considered to be statistically significant.

## 3. Results

### 3.1. Electrophysiologic Effects of the Combination of Pinacidil and Sacubitril

**Figure 3:**
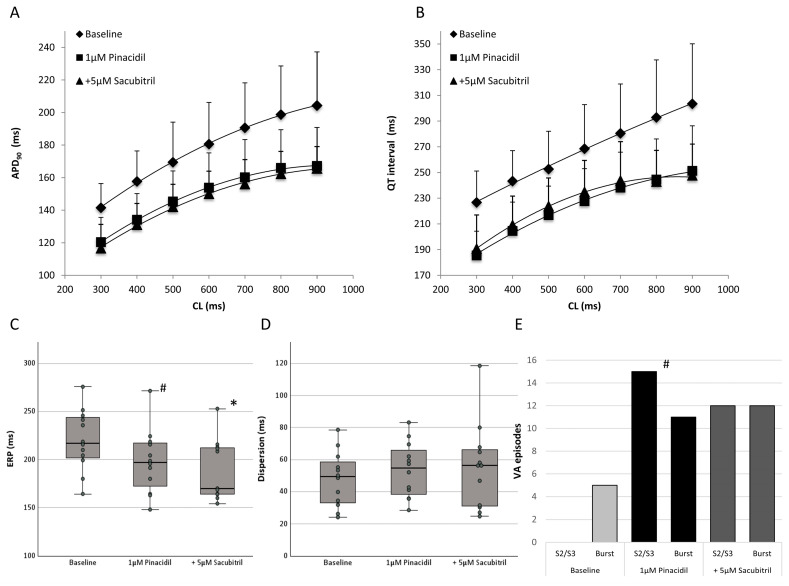
Cycle length-dependent action potential durations at 90% of repolarization ((**A**), APD_90_) and QT interval (**B**) under baseline conditions (◆), after administration of 1 µM pinacidil (■) and after further treatment with 5 µM sacubitril (▲). (**C**,**D**) Box plots showing the impact of 1 µM pinacidil and additive perfusion with 5 µM sacubitril on effective refractory periods (ERP, (**C**)) and spatial dispersion of repolarization (**D**). (**E**) Influence of pinacidil and sacubitril on ventricular arrhythmias (VA). “S2/S3” refers to the induction of arrhythmia through the delivery of premature extra stimuli, while “Burst” indicates the induction of ventricular arrhythmias (VA) through burst pacing. (# = *p* < 0.05 compared to baseline conditions; * = *p* < 0.05 compared to pinacidil).

**Figure 4:**
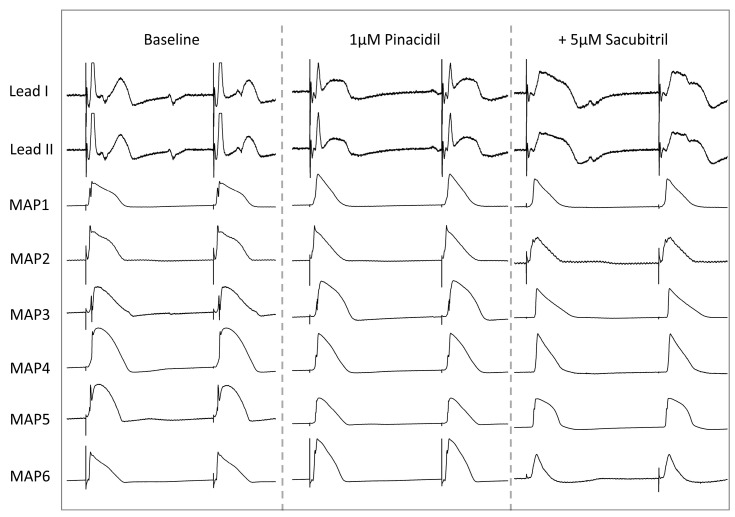
Representative example of ECG and action potential tracings under baseline conditions, after infusion of 1 µM pinacidil and after additional treatment with 5 µM sacubitril. Hearts are stimulated at a cycle length of 900 ms (MAP monophasic action potential).

Infusion of the I_K,ATP_ opener led to a significant reduction in APD_90_ (baseline: 178 ± 25 ms; pinacidil: 150 ± 20 ms, *p* < 0.01; Figure 3A) and QT intervals (baseline: 267 ± 35 ms; pinacidil: 224 ± 26 ms, *p* < 0.01; Figure 3B). Effective refractory periods as determined by programmed ventricular stimulation were also abbreviated (baseline: 220 ± 32 ms; pinacidil: 199 ± 33 ms, *p* < 0.01; Figure 3C), whereas post-repolarization refractoriness was not significantly altered due to the uniform reduction in ERP and APD_90_ (baseline: 43 ± 31 ms; pinacidil: 50 ± 32 ms, *p* = ns). Spatial dispersion of repolarization was not altered in the presence of pinacidil (baseline: 48 ± 20 ms; pinacidil: 54 ± 18 ms, *p* = ns; Figure 3D). Exemplary ECG and MAP tracings are shown in Figure 4.

The additional infusion of sacubitril had only marginal effects on APD_90_ and no significant impact on QT intervals when compared with sole pinacidil treatment (APD_90_: 146 ± 14 ms, *p* < 0.01; QT interval: 228 ± 25 ms, *p* = ns). Refractory periods as well as post-repolarization refractoriness were significantly reduced (ERP: 185 ± 35 ms, *p* < 0.01; PRR: 39 ± 31 ms, *p* < 0.01). Spatial dispersion remained stable (56 ± 29 ms, *p* = ns). Cycle-length dependent changes of APD_90_, QT interval, spatial dispersion of repolarization and ERP are displayed in Table 1.

Under baseline conditions, five episodes of ventricular arrhythmias occurred after burst pacing whereas no arrhythmias were observed when delivering short-coupled extrastimuli (S_2_ and S_3_). In the presence of pincacidil, significantly more ventricular arrhythmias (26 episodes in total; *p* < 0.05 compared to baseline conditions; Figure 3E) were inducible by burst pacing (11 episodes) and delivering closely coupled extrastimuli (15 episodes). After additional treatment with sacubitril, 24 episodes occurred (S_2_/S_3_: 12 episodes; burst pacing: 12 episodes; *p* = ns).

### 3.2. Electrophysiologic Effects of the Combination of Ouabain and Sacubitril

**Figure 5:**
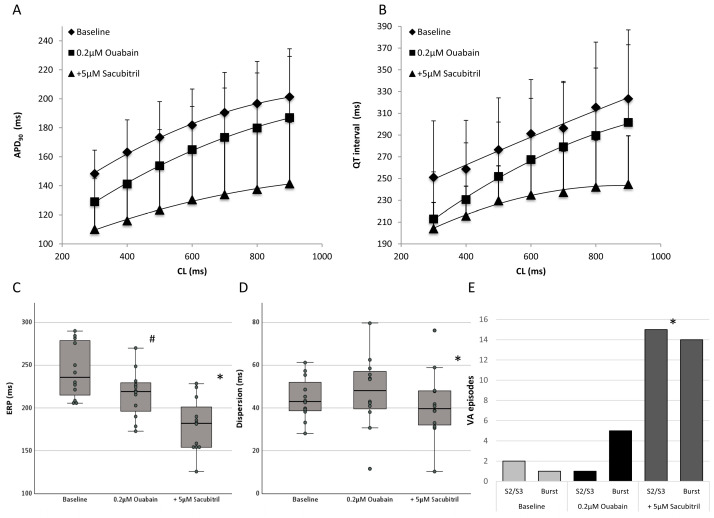
Cycle length-dependent action potential durations at 90% of repolarization ((**A**), APD_90_) and QT interval (**B**) under baseline conditions (◆), after administration of 0.2 µM ouabain (■) and after further treatment with 5 µM sacubitril (▲). (**C**,**D**) Box plots showing the impact of 0.2 µM ouabain and additive perfusion with 5 µM sacubitril on effective refractory periods (ERP, (**C**)) and spatial dispersion of repolarization (**D**). (**E**) Influence of ouabain and sacubitril on ventricular arrhythmias (VA). “S2/S3” refers to the induction of arrhythmia through the delivery of premature extra stimuli, while “Burst” indicates the induction of ventricular arrhythmias (VA) through burst pacing. (# = *p* < 0.05 compared to baseline conditions; * = *p* < 0.05 compared to ouabain).

**Figure 6:**
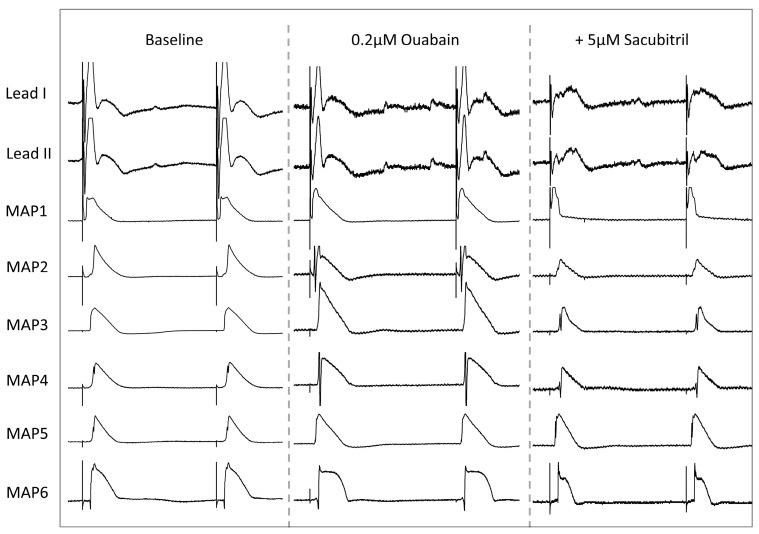
Representative example of ECG and action potential tracings under baseline conditions, after infusion of 0.2 µM ouabaine and after additional treatment with 5 µM sacubitril. Hearts are stimulated at a cycle length of 900 ms (MAP monophasic action potential).

Administration of the digitalis glycoside ouabain (0.2 µM) significantly reduced APD_90_ (baseline: 179 ± 25 ms; ouabain: 161 ± 30 ms, *p* < 0.01; Figure 5A) and QT intervals (baseline: 288 ± 52 ms; ouabain: 262 ± 56 ms, *p* < 0.01; Figure 5B). Effective refractory periods (baseline: 243 ± 39 ms; ouabain: 217 ± 28 ms, *p* < 0.01; Figure 5C), as well as the PRR (baseline: 64 ± 31 ms; ouabain: 55 ± 32 ms, *p* < 0.05), were shortened. Spatial dispersion of repolarization remained stable (baseline: 44 ± 12 ms; ouabain: 48 ± 20 ms, *p* = ns; Figure 5D). Exemplary ECG and MAP tracings are depicted in Figure 6.

Additional infusion of sacubitril substantially shortened cardiac repolarization duration (APD_90_: to 128 ± 33 ms, *p* < 0.01; QT interval: 230 ± 35 ms, *p* < 0.01) and slightly reduced spatial dispersion (41 ± 18 ms, *p* < 0.05). In parallel, effective refractory periods were shortened (179 ± 33 ms, *p* < 0.01) whereas PRR remained stable (52 ± 27 ms, *p* = ns). Cycle-length dependent changes of APD_90_, QT interval, spatial dispersion of repolarization and ERP are displayed in Table 2.

Under baseline conditions, three episodes of ventricular arrhythmias occurred (S_2_/S_3_: two episodes; burst: one episode). Ouabain treatment did not increase the ventricular vulnerability (six episodes in total; S2/S3: one episode; burst: five episodes; *p* = ns; Figure 5E) whereas further infusion of sacubitril was proarrhythmic (29 episodes in total; S2/S3: 15 episodes; burst: 14 episodes; *p* < 0.01).

## 4. Discussion

The present study investigated possible electrophysiological interactions of sacubitril with the two QT interval shortening agents pinacidil and ouabain. The main results are as follows:

(1) Sacubitril mildly shortens APD_90_ in pinacidil-pretreated hearts and significantly reduces effective refractory periods. Further administration of sacubitril did not induce arrhythmias.
(2) In ouabain-pretreated hearts, sacubitril led to a substantial shortening of APD_90_ and QT interval along with a reduction in effective refractory periods.
(3) The repolarization-shortening effect of sacubitril was much more pronounced in ouabain compared to pinacidil-pretreated hearts. This resulted in significantly more ventricular arrhythmias in the presence of additional sacubitril in the ouabain group.

### 4.1. Combination of Pinacidil and Sacubitril

In the present study, pinacidil mimicked the electrophysiologic effects observed in short QT syndrome by activating I_K,ATP_ and thereby reducing cardiac repolarization duration, subsequently facilitating the occurrence of arrhythmias and leading to an increased ventricular vulnerability. Another proarrhythmic mechanism of pinacidil is explained by the inhomogeneous distribution of I_K,ATP_, which is more expressed in the epicardium, thereby creating an inhomogeneous spatial repolarization and a substrate for phase 2 re-entry [23]. These present results are in line with previous studies in which pinacidil was used for the simulation of congenital short QT syndrome [10].

Additional sacubitril treatment had only minor effects on cardiac repolarization duration (reduction in APD_90_ by 3%; no change of QT interval). Of note, the reduction in effective refractory periods is often regarded as the main arrhythmic mechanism in shortened repolarization.

To better contextualize the effects induced by sacubitril, it is essential to consider its sole impact on cardiac electrophysiology. We have previously described the electrophysiologic effects of sacubitril in drug-naïve hearts: In this study [2], administration of 5 µM sacubitril had much more pronounced effects on cardiac repolarization in drug-naïve hearts (reduction in APD_90_ by 24% and of QT interval by 13%). In this prior study [2], 3 µM and 10 µM sacubitril also led to an abbreviation of APD_90_ (reduction by 18% (3 µM) and by 17% (10 µM)) and QT intervals (shortening by 10% (3 µM) and by 5% (10 µM)), though the effects induced by 3 and 10 µM were less pronounced than with 5 µM. Spatial dispersion of repolarization was reduced at each concentration, while the effective refractory periods were not significantly altered. No proarrhythmic effects were observed at any concentration.

In the same study [2], drug-induced long QT syndromes type 2 and 3 were simulated by administration of erythromycin or veratridine, respectively. In the long QT type 2 group, additional sacubitril treatment abbreviated cardiac repolarization duration and reduced spatial dispersion of depolarization. Thereby, sacubitril reduced the occurrence of early afterdepolarizations and torsade de pointes tachycardia. In contrast, further sacubitril treatment did not significantly alter repolarization duration and consequently did not exert antiarrhythmic properties in the long QT syndrome type 3 group.

To summarize, combined treatment with the repolarization shortening agents pinacidil and sacubitril has no significant additive impact on cardiac repolarization duration or arrhythmic risk. These findings are important since, as mentioned above, sacubitril alone has a substantial impact on cardiac electrophysiology. However, when administered in addition to pinacidil-pretreated hearts, sacubitril does not significantly alter repolarization. One possible explanation is that sacubitril may influence ion currents that have already been affected by the pinacidil pretreatment. To be more precise, sacubitril could open I_K,ATP_ channels, which are already activated by pinacidil. However, a previous study demonstrated that sacubitril did not induce endothelial K_ATP_, undermining this possible explanation [24]. In contrast, the combined administration of different QT-prolonging agents results in an additive lengthening of cardiac repolarization and ultimately in a relevant arrhythmic risk [25]. Other potential mechanisms underlying the sacubitril-induced shortening of repolarization include enhanced I_Ks_ or I_Kr_, or reduced I_Na,L_ or I_Ca_. Notably, these are the major currents that influence the proarrhythmic risk and are therefore considered in The Comprehensive in Vitro Proarrhythmia Assay (CiPA) initiative for assessing the safety of new drugs [26]. It is likely that sacubitril does not exert ion channel-specific effects, but instead has complex effects on cardiac ion channels. Since no experimental studies have been conducted to further elucidate this, these hypotheses remain speculative [2].

Notably, cardiac K_ATP_ channels are inhibited by physiological intracellular ATP levels, linking cellular metabolism with membrane potential [27]. As a result, ischemia activates I_K,ATP_ and thereby abbreviates action potential duration and decreases I_Ca,L_, thus protecting the cell from calcium overload and enhancing cell survival. It is important to note that the shortening of the action potential during acute ischemia primarily occurs early in the plateau phase [28,29]. These pathophysiological effects underscore the crucial role of I_K,ATP_ activation in ischemic preconditioning [27]. However, the downside is that K_ATP_ activation, in addition to shortening the action potential, leads to the accumulation of extracellular K^+^ and a reduced conduction velocity, which can facilitate re-entrant ventricular tachycardias [27]. Another proarrhythmic mechanism is attributed to the inhomogeneous distribution of I_K,ATP_, which is more pronounced in the epicardium. This creates a heterogeneous repolarization, providing a substrate for phase 2 re-entry [23]. Due to these effects, pinacidil is commonly used to simulate electrophysiological conditions during ischemia [23]. In this light, sacubitril exerts a safe electrophysiologic profile in a model of acute ischemia, lacking proarrhythmic effects despite its repolarization-shortening properties.

### 4.2. Combination of Ouabain and Sacubitril

Treatment with ouabain also reduced cardiac repolarization duration and effective refractory periods. There are different potential mechanisms that might contribute to the action potential shortening effect of digitalis glycosides: (1) Digitalis glycosides inhibit the Na^+^-K^+^-ATPase, augmenting the intracellular sodium and calcium concentration and thereby increasing potassium permeability. This in turn increases the outward current during the plateau phase and abbreviates the action potential [12]. (2) Increased levels of cytosolic calcium accelerate calcium-dependent inactivation of the L-type calcium channel, reducing I_Ca,L_ and thereby shortening action potential duration [30].

No significant proarrhythmic effects were observed in the present study under ouabain infusion alone. Additional administration of sacubitril had a significant impact on cardiac repolarization as APD_90_ was abbreviated by 20%, QT interval by 13% and ERP by 18%. The underlying mechanism for these effects may involve an increase in I_Kr_, I_Ks_, or I_K,ATP_, or a decrease in I_Na,L_ or I_Ca_. The shortening of cardiac repolarization duration, and particularly the refractory periods, can promote re-entry and potentially explain the increased occurrence of ventricular arrhythmias in the presence of sacubitril. The abbreviation of ventricular refractoriness facilitates re-entry by shortening the wavelength of the re-entry circuit [31].

However, it may be too simplistic to attribute the arrhythmic risk solely to the abbreviation of cardiac repolarization duration. There are other mechanisms that contribute to arrhythmias with digitalis glycosides such as an amplification of autonomic activity [32], which can be ignored in this study due to the experimental setup. In addition, digitalis glycosides can trigger delayed afterdepolarizations by spontaneous calcium release from the sarcoplasmatic reticulum due to calcium overload and CaMKII-mediated modifications of the ryanodine receptor [33] and thus promote polymorphic ventricular tachycardias [13,14]. The observations from this study are not the first to suggest an increased arrhythmic risk when combining digitalis glycosides with other drugs that influence cardiac electrophysiology. The PALLAS trial (Permanent Atrial Fibrillation Outcome Study Using Dronedarone on Top of Standard Therapy) [34] demonstrated that dronedarone increased the risk of death from cardiovascular causes in patients with permanent atrial fibrillation who were at risk for major vascular events. This adverse effect was at least in part driven by arrhythmic events under comedication with digoxin [35] and can be attributed to higher digoxin serum levels on dronedarone and a substantial abbreviation of cardiac repolarization [15].

### 4.3. Limitations

This study was conducted employing a whole-heart setup. Therefore, this model does not permit precise conclusions regarding the direct effects on single ion channels. However, the Langendorff-perfused rabbit heart model is well-established for studying drug-induced proarrhythmia due to its similarity in the distribution of repolarizing ion currents [36].

The hearts were perfused only with sacubitril, not with the clinically employed combination of sacubitril and valsartan. Consequently, one could assume that combining sacubitril with valsartan might induce different electrophysiological effects, which could limit the significance of the present study. However, a previous study demonstrated that valsartan alone, at clinically relevant concentrations, has no significant impact on either ventricular or atrial action potential duration (APD_90_) in rat and guinea pig hearts [37]. Given that valsartan did not exhibit substantial effects on myocardial electrophysiology, we did not expect any additional acute electrophysiological effects when combining sacubitril and valsartan.

## 5. Conclusions

Sacubitril has a divergent electrophysiologic profile in pinacidil- and digitalis-related shortened repolarization. Sacubitril does not increase the arrhythmic risk in pinacidil-pre-treated hearts. Thus, the sole combination of QT interval-shortening drugs itself does not harbor an exceeding proarrhythmic risk as it can be observed when combining QT-prolonging drugs [25]. However, sacubitril administration led to an increased arrhythmic risk in hearts already perfused with ouabain. Thus, the combination of sacubitril and digitalis glycosides requires careful consideration.

## Figures and Tables

**Table 1:** Cycle length-dependent action potential durations (APD_90_), QT intervals, spatial dispersion of repolarization and effective refractory periods (ERP) under baseline conditions, with pinacidil and after additional treatment with sacubitril.

	Cycle Length	Baseline	1 µM Pinacidil	5 µM Sacubitril
APD_90_	900	204 ± 33	167 ± 24	166 ±13
	800	199 ± 30	166 ± 23	162 ± 14
	700	191 ± 28	160 ± 23	156 ± 15
	600	181 ± 26	154 ± 21	150 ± 14
	500	169 ± 25	145 ± 19	142 ± 14
	400	158 ± 19	134 ± 16	131 ± 13
	300	141 ± 15	120 ± 15	117 ± 15
QT	900	304 ± 47	251 ± 35	248 ± 24
	800	293 ± 45	245 ± 32	243 ± 24
	700	281 ± 38	238 ± 28	244 ± 30
	600	269 ± 34	228 ± 25	235 ± 24
	500	253 ± 29	217 ± 23	224 ± 22
	400	243 ± 24	205 ± 22	209 ± 22
	300	227 ± 24	186 ± 19	191 ± 26
Dispersion	900	51 ± 21	58 ± 19	60 ± 34
	800	53 ± 24	59 ± 21	57 ± 33
	700	51 ± 22	55 ± 20	57 ± 30
	600	50 ± 20	53 ± 18	54 ± 32
	500	48 ± 17	52 ± 16	57 ± 23
	400	43 ± 16	50 ± 15	52 ± 20
	300	41 ± 16	49 ± 17	57 ± 29
ERP	900	228 ± 31	210 ± 30	192 ± 30
	800	226 ± 32	207 ± 30	190 ± 34
	700	225 ± 32	204 ± 32	188 ± 36
	600	223 ± 32	200 ± 33	186 ± 39
	500	218 ± 31	198 ± 35	185 ± 40
	400	214 ± 30	193 ± 35	181 ± 37
	300	208 ± 35	186 ± 36	176 ± 36

**Table 2:** Cycle length-dependent action potential durations (APD_90_), QT intervals, spatial dispersion of repolarization and effective refractory periods (ERP) under baseline conditions, with ouabain and after additional treatment with sacubitril.

	Cycle Length	Baseline	0.2 µM Ouabain	5 µM Sacubitril
APD_90_	900	201 ± 33	187 ± 42	141 ± 42
	800	197 ± 29	180 ± 38	138 ± 41
	700	190 ± 28	173 ± 34	134 ± 38
	600	182 ± 25	165 ± 30	130 ± 33
	500	173 ± 25	154 ± 25	123 ± 29
	400	163 ± 22	141 ± 22	116 ± 26
	300	148 ± 16	129 ± 16	110 ± 21
QT	900	323 ± 63	302 ± 71	245 ± 45
	800	316 ± 60	290 ± 62	242 ± 43
	700	296 ± 43	279 ± 59	237 ± 38
	600	291 ± 50	267 ± 56	235 ± 34
	500	277 ± 48	252 ± 50	230 ± 32
	400	259 ± 45	231 ± 52	216 ± 27
	300	251 ± 52	213 ± 43	204 ± 24
Dispersion	900	50 ± 11	53 ± 28	39 ± 19
	800	48 ± 10	54 ± 23	39 ± 17
	700	45 ± 10	48 ± 20	41 ± 16
	600	45 ± 14	47 ± 18	40 ± 18
	500	40 ± 12	45 ± 17	44 ± 16
	400	40 ± 13	45 ± 18	44 ± 17
	300	42 ± 15	41 ± 17	41 ± 20
ERP	900	258 ± 38	221 ± 25	175 ± 37
	800	259 ± 42	223 ± 27	179 ± 34
	700	256 ± 41	223 ± 30	179 ± 35
	600	251 ± 38	222 ± 28	179 ± 32
	500	236 ± 29	216 ± 30	180 ± 32
	400	228 ± 34	210 ± 28	179 ± 34
	300	217 ± 37	203 ± 31	184 ± 36

## Data Availability

The datasets generated during and analysed during the current study are available from the corresponding author on reasonable request.

## References

[1] McDonagh T.A., Metra M., Adamo M., Gardner R.S., Baumbach A., Böhm M., Burri H., Butler J., Čelutkienė J., Chioncel O. (2021). 2021 ESC Guidelines for the diagnosis and treatment of acute and chronic heart failure: Developed by the Task Force for the diagnosis and treatment of acute and chronic heart failure of the European Society of Cardiology (ESC) With the special contribution of the Heart Failure Association (HFA) of the ESC. Eur. Heart J..

[2] Ellermann C., Dimanski D., Wolfes J., Rath B., Leitz P., Willy K., Wegner F.K., Eckardt L., Frommeyer G. (2022). Electrophysiologic effects of sacubitril in different arrhythmia models. Eur. J. Pharmacol..

[3] Weir R.A. (2019). Sacubitril/Valsartan and Mexiletine: A Proarrhythmic Combination?. Cardiology.

[4] Gatti M., Antonazzo I.C., Diemberger I., De Ponti F., Raschi E. (2021). Adverse events with sacubitril/valsartan in the real world: Emerging signals to target preventive strategies from the FDA adverse event reporting system. Eur. J. Prev. Cardiol..

[5] Gussak I., Brugada P., Brugada J., Wright R.S., Kopecky S.L., Chaitman B.R., Bjerregaard P. (2001). Idiopathic short QT interval: A new clinical syndrome?. Cardiology.

[6] Nielsen J.C., Lin Y.-J., de Oliveira Figueiredo M.J., Sepehri Shamloo A., Alfie A., Boveda S., Dagres N., Di Toro D., Eckhardt L.L., Ellenbogen K. (2020). European Heart Rhythm Association (EHRA)/Heart Rhythm Society (HRS)/Asia Pacific Heart Rhythm Society (APHRS)/Latin American Heart Rhythm Society (LAHRS) expert consensus on risk assessment in cardiac arrhythmias: Use the right tool for the right outcome, in the right population. Europace.

[7] Iribarren C., Round A.D., Peng J.A., Lu M., Klatsky A.L., Zaroff J.G., Holve T.J., Prasad A., Stang P. (2014). Short QT in a cohort of 1.7 million persons: Prevalence, correlates, and prognosis. Ann. Noninvasive Electrocardiol..

[8] Shah R.R. (2010). Drug-induced QT interval shortening: Potential harbinger of proarrhythmia and regulatory perspectives. Br. J. Pharmacol..

[9] Bjerregaard P., Gussak I. (2005). Short QT syndrome: Mechanisms, diagnosis and treatment. Nat. Clin. Pract. Cardiovasc. Med..

[10] Milberg P., Tegelkamp R., Osada N., Schimpf R., Wolpert C., Breithardt G., Borggrefe M., Eckardt L. (2007). Reduction of dispersion of repolarization and prolongation of postrepolarization refractoriness explain the antiarrhythmic effects of quinidine in a model of short QT syndrome. J. Cardiovasc. Electrophysiol..

[11] Cheng T.O. (2004). Digitalis administration: An underappreciated but common cause of short QT interval. Circulation.

[12] Garberoglio L., Giustetto C., Wolpert C., Gaita F. (2007). Is acquired short QT due to digitalis intoxication responsible for malignant ventricular arrhythmias?. J. Electrocardiol..

[13] Rosen M.R. (1985). Cellular electrophysiology of digitalis toxicity. J. Am. Coll. Cardiol..

[14] Ellermann C., Wolfes J., Puckhaber D., Bögeholz N., Leitz P., Lange P.S., Eckardt L., Frommeyer G. (2019). Digitalis Promotes Ventricular Arrhythmias in Flecainide-and Ranolazine-Pretreated Hearts. Cardiovasc. Toxicol..

[15] Frommeyer G., Milberg P., Schulze Grotthoff J., Dechering D.G., Kochhäuser S., Stypmann J., Fehr M., Breithardt G., Eckardt L. (2015). Dronedarone and digitalis: Individually reduced post-repolarization refractoriness enhances life-threatening arrhythmias. Europace.

[16] Eisen A., Ruff C.T., Braunwald E., Hamershock R.A., Lewis B.S., Hassager C., Chao T.F., Le Heuzey J.Y., Mercuri M., Rutman H. (2017). Digoxin Use and Subsequent Clinical Outcomes in Patients With Atrial Fibrillation With or Without Heart Failure in the ENGAGE AF-TIMI 48 Trial. J. Am. Heart Assoc..

[17] Washam J.B., Stevens S.R., Lokhnygina Y., Halperin J.L., Breithardt G., Singer D.E., Mahaffey K.W., Hankey G.J., Berkowitz S.D., Nessel C.C. (2015). Digoxin use in patients with atrial fibrillation and adverse cardiovascular outcomes: A retrospective analysis of the Rivaroxaban Once Daily Oral Direct Factor Xa Inhibition Compared with Vitamin K Antagonism for Prevention of Stroke and Embolism Trial in Atrial Fibrillation (ROCKET AF). Lancet.

[18] Friedel H.A., Brogden R.N. (1990). Pinacidil: A review of its pharmacodynamic and pharmacokinetic properties, and therapeutic potential in the treatment of hypertension. Drugs.

[19] Wolfes J., Uphoff J., Kemena S., Wegner F., Rath B., Eckardt L., Frommeyer G., Ellermann C. (2024). Divergent electrophysiologic action of dapagliflozin and empagliflozin on ventricular and atrial tachyarrhythmias in isolated rabbit hearts. Front. Cardiovasc. Med..

[20] De Pover A., Godfraind T. (1979). Interaction of ouabain with (Na++ K+) ATPase from human heart and from guinea-pig heart. Biochem. Pharmacol..

[21] Palasis M., Kuntzweiler T.A., Argüello J.M., Lingrel J.B. (1996). Ouabain interactions with the H5-H6 hairpin of the Na, K-ATPase reveal a possible inhibition mechanism via the cation binding domain. J. Biol. Chem..

[22] Kobalava Z., Kotovskaya Y., Averkov O., Pavlikova E., Moiseev V., Albrecht D., Chandra P., Ayalasomayajula S., Prescott M.F., Pal P. (2016). Pharmacodynamic and Pharmacokinetic Profiles of Sacubitril/Valsartan (LCZ 696) in Patients with Heart Failure and Reduced Ejection Fraction. Cardiovasc. Ther..

[23] Di Diego J., Antzelevitch C. (1993). Pinacidil-induced electrical heterogeneity and extrasystolic activity in canine ventricular tissues. Does activation of ATP-regulated potassium current promote phase 2 reentry?. Circulation.

[24] Seki T., Goto K., Kansui Y., Ohtsubo T., Matsumura K., Kitazono T. (2017). Angiotensin II receptor–neprilysin inhibitor sacubitril/valsartan improves endothelial dysfunction in spontaneously hypertensive rats. J. Am. Heart Assoc..

[25] Frommeyer G., Fischer C., Ellermann C., Dechering D.G., Kochhäuser S., Lange P.S., Wasmer K., Fehr M., Eckardt L. (2018). Additive Proarrhythmic Effect of Combined Treatment with QT-Prolonging Agents. Cardiovasc. Toxicol..

[26] Sager P.T., Gintant G., Turner J.R., Pettit S., Stockbridge N. (2014). Rechanneling the cardiac proarrhythmia safety paradigm: A meeting report from the Cardiac Safety Research Consortium. Am. Heart J..

[27] Tamargo J., Caballero R., Gómez R., Valenzuela C., Delpón E. (2004). Pharmacology of cardiac potassium channels. Cardiovasc. Res..

[28] Shaw R.M., Rudy Y. (1997). Electrophysiologic effects of acute myocardial ischemia: A theoretical study of altered cell excitability and action potential duration. Cardiovasc. Res..

[29] Cole W.C. (1993). ATP-sensitive K+ channels in cardiac ischemia: An endogenous mechanism for protection of the heart. Cardiovasc. Drugs Ther..

[30] Koh C.H., Wu J., Chung Y.Y., Liu Z., Zhang R.-R., Chong K., Korzh V., Ting S., Oh S., Shim W. (2017). Identification of Na+/K+-ATPase inhibition-independent proarrhythmic ionic mechanisms of cardiac glycosides. Sci. Rep..

[31] Waks J.W., Josephson M.E. (2014). Mechanisms of atrial fibrillation–reentry, rotors and reality. Arrhythm. Electrophysiol. Rev..

[32] Hauptman P.J., Kelly R.A. (1999). Digitalis. Circulation.

[33] Gonano L.A., Petroff M.V. (2014). Subcellular mechanisms underlying digitalis-induced arrhythmias: Role of calcium/calmodulin-dependent kinase II (CaMKII) in the transition from an inotropic to an arrhythmogenic effect. Heart Lung Circ..

[34] Connolly S.J., Camm A.J., Halperin J.L., Joyner C., Alings M., Amerena J., Atar D., Avezum Á., Blomström P., Borggrefe M. (2011). Dronedarone in high-risk permanent atrial fibrillation. N. Eng. J. Med..

[35] Hohnloser S.H., Halperin J.L., Camm A.J., Gao P., Radzik D., Connolly S.J. (2014). Interaction between digoxin and dronedarone in the PALLAS trial. Circ. Arrhythm. Electrophysiol..

[36] Clauss S., Bleyer C., Schüttler D., Tomsits P., Renner S., Klymiuk N., Wakili R., Massberg S., Wolf E., Kääb S. (2019). Animal models of arrhythmia: Classic electrophysiology to genetically modified large animals. Nat. Rev. Cardiol..

[37] Cox M., de Gasparo M., Mukherjee R., Hewett K., Spinale F. (1997). Myocardial electrophysiological properties in the presence of an AT 1 angiotensin II receptor antagonist. Basic. Res. Cardiol..

